# Reduction of molecular oxygen by redox active thiols: comparison of glutathione, *N*-acetylcysteine, cysteine, and homocysteine

**DOI:** 10.3164/jcbn.19-25

**Published:** 2019-09-11

**Authors:** Minako Nyui, Yoshimi Shoji, Megumi Ueno, Ikuo Nakanishi, Ken-ichiro Matsumoto

**Affiliations:** 1 Quantitative RedOx Sensing Group, Department of Basic Medical Sciences for Radiation Damages, National Institute of Radiological Sciences, Quantum Medical Science Directorate, National Institutes for Quantum and Radiological Science and Technology, 4-9-1 Anagawa, Inage-ku, Chiba 263-8555, Japan; 2 Quantum-state Controlled MRI Group, Institute for Quantum Life Science, National Institutes for Quantum and Radiological Science and Technology, 4-9-1 Anagawa, Inage-ku, Chiba 263-8555, Japan

**Keywords:** thiol, reductive stress, reactive sulfur species, nitroxide, electron paramagnetic resonance

## Abstract

The reaction properties of the thiol compounds, cysteine (Cys), *N*-acetyl-l-cysteine (NAC), the reduced form glutathione (GSH), and homocysteine (HCS) were compared. The main purpose of this study was to find a thiol-based anti-oxidant suitable for biological experiments and to provide clear reasoning for its selection. The availability of thiol compounds to generate superoxide by reducing molecular oxygen (O_2_) at a hyperthermal temperature was discussed. An oxidative atmosphere, i.e., superoxide generation by the hypoxanthine-xanthine oxidase reaction, hydroxyl radical generation by X-ray irradiation, or direct one-electron oxidation by ferricyanide, was prepared in a reaction mixture containing 0.1 mM TEMPOL and 1 mM test compound, and the EPR signal decay of TEMPOL was observed. A reaction mixture containing 0.1 mM TEMPOL and 1 mM thiol compound was incubated at 44°C, and the EPR signal decay of TEMPOL was observed. Thiols could function as H-donors to the oxoammonium cation and produce the hydroxylamine form of TEMPOL in an oxidative atmosphere. Thiols could also irreversibly react with the oxoammonium cation. GSH and Cys could reduce O_2_ to form superoxide/hydroperoxyl radical at hyperthermal temperatures, but HCS and NAC could not reduce O_2_. GSH and Cys may cause reductive stress, whereas NAC is a simple tractable antioxidant.

## Introduction

**Fig. 1:**
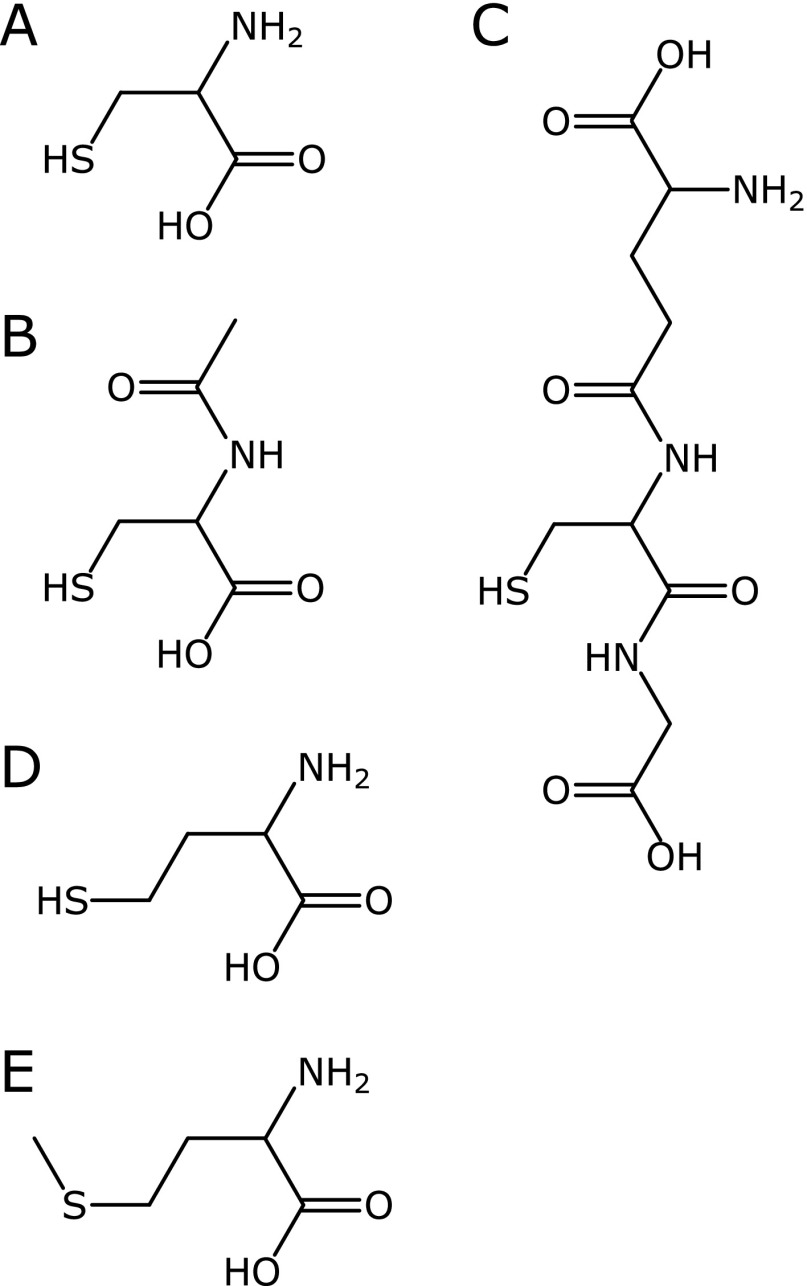
Chemical structures of sulfur-containing compounds compared in this study. (A) Cys is a simple amino acid. (B) NAC is an acetylated derivative of Cys. (C) GSH is a tripeptide, consisting of glutamic acid, Cys, and glycine. (D) HCS is homologous to Cys. (E) Met is a methylated derivative of HCS.

Several thiol compounds are widely used as anti-oxidants in biological experiments. For example, cysteine (Cys) (Fig. 1A) is a relatively simple amino acid that has a thiol moiety. *N*-Acetyl-l-cysteine (NAC) (Fig. 1B) is a derivative of Cys acetylated on the amino moiety. The main endogenous antioxidant in living cells is the reduced form of glutathione (GSH) (Fig. 1C), which is a tripeptide molecule consisting of glutamic acid, Cys, and glycine. Although homocysteine (HCS) has an extra methylene bridge compared with the structure of cysteine (Fig. 1D), HCS may be an unorthodox thiol compound in a negative sense because it was reported to be an inducer of oxidative stress.^(1–3)^ Zhang *et al.*^(4)^ reported that the cerebrovascular effects of HCS were prevented by superoxide dismutase (SOD). HCS is biologically synthesized from methionine (Met), which is an essential amino acid for humans. Met has an extra methyl terminal on the structure of HCS (Fig. 1E).

Superoxide (O_2_^•−^) generation in an aqueous solution containing GSH at a hyperthermal temperature was reported.^(5,6)^ GSH can reduce oxygen (O_2_) to make hydroperoxyl radicals (HO_2_^•^), which equilibrate to O_2_^•−^ in an aqueous environment. HO_2_^•^ is a more efficient oxidant than O_2_^•−^, which is essentially a reductant. The GSH-induced HO_2_^•^/O_2_^•−^ redox reaction at hyperthermal temperatures can cause cell death.^(5)^ The excellent biological reductant GSH can reduce oxygen to give ROS and cause cell toxicity at hyperthermal temperatures. The high reducing activity of anti-oxidants may cause oxidative stress.

This GSH-induced cell death at hyperthermal temperatures was amplified by the addition of catalase.^(5)^ HO_2_^•^ molecules can react together to form hydrogen peroxide (H_2_O_2_). In addition, HO_2_^•^ can be reduced by GSH to give H_2_O_2_. The simultaneously generated H_2_O_2_ by-product of GSH-induced HO_2_^•^/O_2_^•−^ can absorb oxidative stress induced by HO_2_^•^/O_2_^•−^ because it can consume O_2_^•−^. Hydroxyl radicals (^•^OH) may be generated by the reaction of H_2_O_2_ and O_2_^•−^, whereas this extracellular single-shot ^•^OH may have no particular biological effect. As a result, the elimination of H_2_O_2_ by catalase may increase the oxidative stress induced in a HO_2_^•^/O_2_^•−^ atmosphere. Anti-oxidative cooperation in an oxygenated atmosphere can cause further oxidative stress, contrary to expectation, which is termed reductive stress.

The stable nitroxyl radical 4-hydroxyl-2,2,6,6-tetramethylpiperidine-*N*-oxyl (TEMPOL) can be oxidized to an oxoammonium cation form by HO_2_^•^ and/or ^•^OH.^(7,8)^ The oxoammonium cations are reduced to hydroxylamine by accepting a hydrogen atom from hydrogen donors (H-donors), such as reduced GSH, reduced β-nicotinamide adenine dinucleotide (NADH), or reduced β-nicotinamide adenine dinucleotide phosphate (NADPH), in biological settings.^(9–11)^ The oxoammonium cations can also react with GSH directly to make a binding complex.^(12,13)^ TEMPOL can be reduced by two methods with coexisting GSH. Other thiol compounds are also expected to react with TEMPOL in two manners, similar to GSH. However, no confirmation of the reaction of other thiols with TEMPOL or the corresponding oxoammonium form has been reported.

Some biological thiol compounds, such as GSH and/or cysteine, exhibit unpredictable reactions under biological experimental conditions. There are several reports of oxidative stress by thiols, especially by GSH as an oxidant.^(14–20)^ The purpose of this study was to find a suitable thiol-based anti-oxidant for biological experiments and to provide clear reasoning for its selection. In this study, the reaction properties of Cys, NAC, GSH, HCS, and Met were compared using TEMPOL as a redox probe. Furthermore, the availability of thiol compounds to cause reductive stress was discussed.

## Materials and Methods

### Chemicals

GSH, Cys, NAC, HCS, Met, and potassium ferricyanide were purchased from Wako Chemical (Tokyo, Japan). TEMPOL, SOD from human erythrocytes, hypoxanthine, xanthine oxidase, NADH, and NADPH were purchased from Sigma-Aldrich (St. Louis, MO). Other chemicals used were of analytical grade. As basic solvents for the reaction mixtures, 100 mM phosphate buffer (PB) containing 0.05 mM DTPA adjusted to pH 7.4 was prepared. Deionized water (deionization by the Milli-Q system) was used to prepare PB.

### Reaction of TEMPOL with coexisting thiol compounds in the hypoxanthine-xanthine oxidase (HX-XO) reaction system

A reaction mixture containing xanthine oxidase, TEMPOL, and one of the test compounds was prepared using PB. Then, an aliquot of hypoxanthine solution was added to the reaction mixture to start the reaction. The final concentrations of hypoxanthine, xanthine oxidase, TEMPOL, and the test compound were 0.05 mM, 0.01 U/ml, 0.1 mM, and 1.0 mM, respectively. The reaction mixture was drawn into a quartz flat cell, and then fixed in the TE-mode cavity. The time course of the EPR signal intensity of TEMPOL was measured using an X-band EPR spectrometer (JEOL, Tokyo). The EPR conditions were follows: the microwave frequency was 9.4 GHz, microwave power was 4 mW, center field was 334 mT, sweep width was 10 mT, sweep speed was 5 mT/min, modulation frequency was 100 kHz, modulation amplitude was 0.079 mT, and the time constant was 0.03 s. This experiment was performed at room temperature.

### Reaction of TEMPOL with coexisting thiol compounds during X-ray irradiation

A reaction mixture containing 0.1 mM TEMPOL and 1.0 mM test compound was prepared using PB. Then, the reaction mixture was kept on ice until X-ray irradiation. X-ray irradiation was performed using PANTAK 320 (Shimadzu, Kyoto, Japan). The effective energy was 80 keV under the following conditions: the X-ray tube voltage was 200 kV, X-ray tube current was 20 mA, and the thickness and materials of the pre-filter were 0.5-mm copper and 0.5-mm aluminum. The dose rate of X-ray irradiation was 3.3 Gy/min when the distance between the X-ray tube and the sample was 30 cm. The irradiated reaction mixtures were again kept on ice to halt the temperature-dependent reaction until EPR measurements were performed. Dose responses of the EPR signal intensity of TEMPOL were measured using an X-band EPR spectrometer (JEOL, Tokyo) after X-ray irradiation doses of 0, 2, 4, 8, 16, or 32 Gy.

### Reaction of TEMPOL with coexisting thiol compounds in a ferricyanide reaction system

A reaction mixture containing 0.1 mM TEMPOL and 1.0 mM test compound was prepared using PB. Then, a 1/100 volume of 200 mM potassium ferricyanide, K_3_[Fe(CN)_6_], was added to an aliquoted volume of the reaction mixture to start the reaction. The time course of the EPR signal intensity of TEMPOL was measured using an X-band EPR spectrometer. The EPR conditions were as described above. The same experiment was performed adding 20 U/ml SOD to the reaction mixture or by bubbling the reaction mixture with N_2_ gas. In addition, the same experiment was performed using ferric chloride (FeCl_3_) instead of K_3_[Fe(CN)_6_].

### Reduction of ferricyanide by a thiol compound or NAD(P)H

The solutions of K_3_[Fe(CN)_6_] and a test compound were prepared using PB. The K_3_[Fe(CN)_6_] solution and a test compound solution were mixed to make the final concentrations 2 mM and 1 mM, respectively. The time course of absorption at 420 nm of K_3_[Fe(CN)_6_] was observed on an Agilent 8453 photodiode array spectrophotometer (NAD(P)H) or a UNISOKU RSP-1000-02NM spectrophotometer (thiol compounds). The same experiments were repeated with 0.1 mM TEMPOL. For the oxidation of NAD(P)H by ferricyanide, time courses of the absorption at 340 nm after mixing the solutions were also observed.

### Reaction of TEMPOL with coexisting thiol compounds at a hyperthermal temperature

A reaction mixture containing 0.1 mM TEMPOL and 1.0–8.0 mM test compound was prepared using PB. Then, the reaction mixture was incubated at 44°C, and the time course of the EPR signal intensity of TEMPOL in the reaction mixture was measured. The EPR conditions were the same as described above. For GSH and Cys, the same experiment was repeated adding 5 U/ml SOD or by bubbling N_2_ gas into the reaction mixture.

## Results and Discussion

**Fig. 2:**
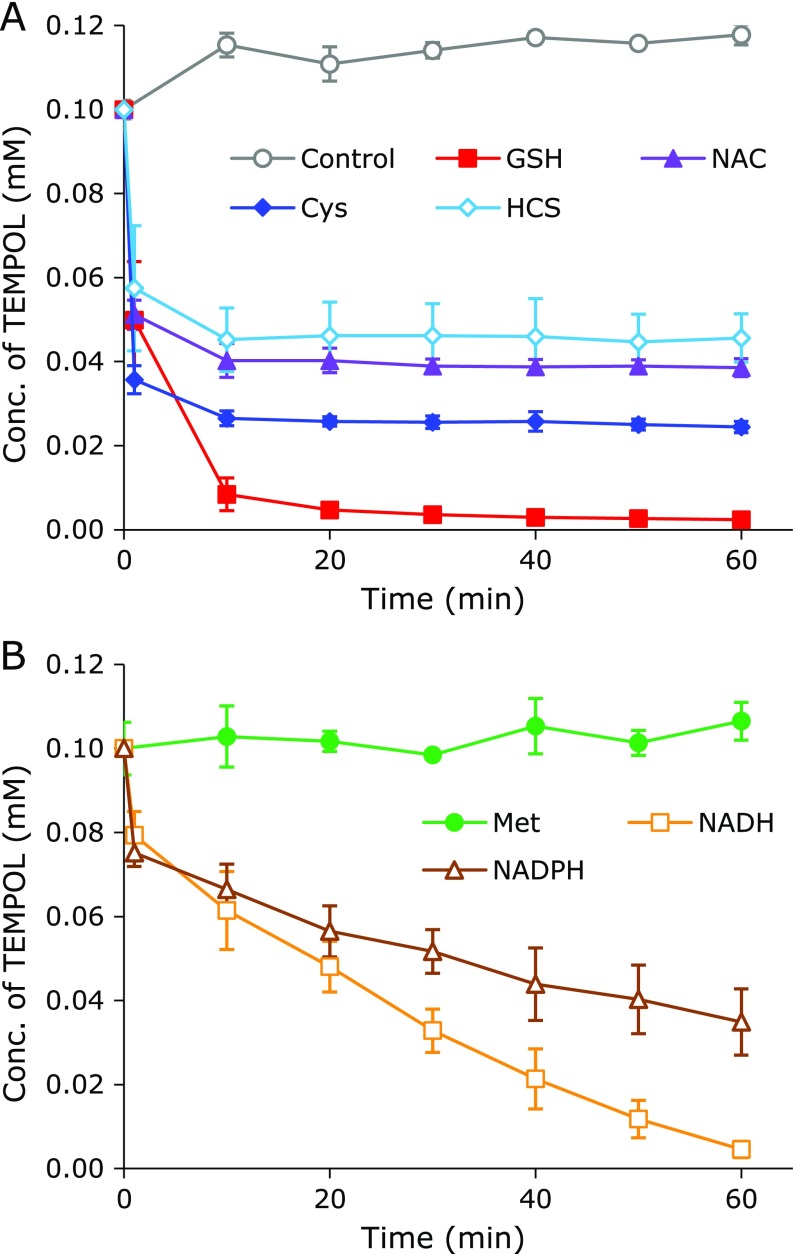
Time course of TEMPOL in the HX-XO reaction system with a coexisting test compound. (A) TEMPOL with a thiol compound, open circle: TEMPOL alone, closed square: GSH, closed triangle: NAC, and closed diamond: Cys, open diamond: HCS. (B) TEMPOL with a non-thiol compound, closed circle: Met, open square: NADH, and open triangle: NADPH. The data points and error bars indicate the average ± SD of 3 experiments. The concentrations of TEMPOL and the test compound were 0.1 mM and 1.0 mM, respectively.

TEMPOL was not reduced when it was exposed alone to the HX-XO reaction system, which induced O_2_^•−^ (Fig. 2A, open circle). The O_2_^•−^ equilibrated with HO_2_^•^ in an aqueous environment. HO_2_^•^, which is a strong oxidant, can one-electron-oxidize the nitroxyl radical form of TEMPOL to produce the corresponding oxoammonium cation form. The oxoammonium cation can be one-electron-reduced by O_2_^•−^, which is basically a reductant, to produce the nitroxyl radical form again. Therefore, TEMPOL was unaffected in the HX-OX reaction system. The oxoammonium cation form of TEMPOL can be two-electron-reduced to produce a relatively stable hydroxylamine form when it receives a hydrogen atom (^•^H) from a coexisting hydrogen donor compound. The oxoammonium cation form of TEMPOL can also directly bind to a thiol moiety to make an EPR-silent complex. The reduction profile of TEMPOL in the HX-XO reaction system with a coexisting thiol compound is shown in Fig. 2A. All thiol compounds tested, including GSH (closed square), NAC (closed triangle), Cys (closed diamond), and HCS (open diamond), were able to reduce TEMPOL in the HX-OX reaction system. However, Met (Fig. 2B, closed circle), a sulfur-containing amino acid lacking a thiol, did not function as a reductant for the oxoammonium cation. NADH (Fig. 2B, open square) and NADPH (Fig. 2B, open triangle) functioned as a hydrogen donor, and reduced TEMPOL in the HX-OX reaction system.

The HX-OX reaction system used in this experiment continued generating HO_2_^•^/O_2_^•−^ during the 60-min experimental period because the TEMPOL with coexisting NADH or NADPH continued reducing. The incomplete elimination of TEMPOL with coexisting NAC, Cys, or HCS in this HX-OX reaction system may have been due to depletion of the thiol compounds by HO_2_^•^, even though 10-times more thiol compound than TEMPOL was added to the reaction mixture. Thus, most thiols can be oxidized by HO_2_^•^ and be consumed before reducing the oxoammonium cation. However, TEMPOL was eliminated with higher concentrations of NAC, Cys, or HCS (data not shown), excluding excess Cys (8 mM), which inhibited complete TEMPOL reduction (data not shown). The oxoammonium cation form of TEMPOL and HO_2_^•^ compete to oxidize thiols. Therefore, the EPR signal loss of TEMPOL was suppressed when the reaction of HO_2_^•^ and a thiol compound was faster than the reaction of the oxoammonium cation and a thiol. The free radical form of TEMPOL and thiols may also simultaneously compete with HO_2_^•^. If this is the case, the EPR signal loss of TEMPOL may again be suppressed when the HO_2_^•^-induced oxidation of a thiol compound is faster than the oxidation of TEMPOL. As such, the relative reduction ability of thiols by HO_2_^•^ may be Cys > HCS > NAC > GSH.

**Fig. 3:**
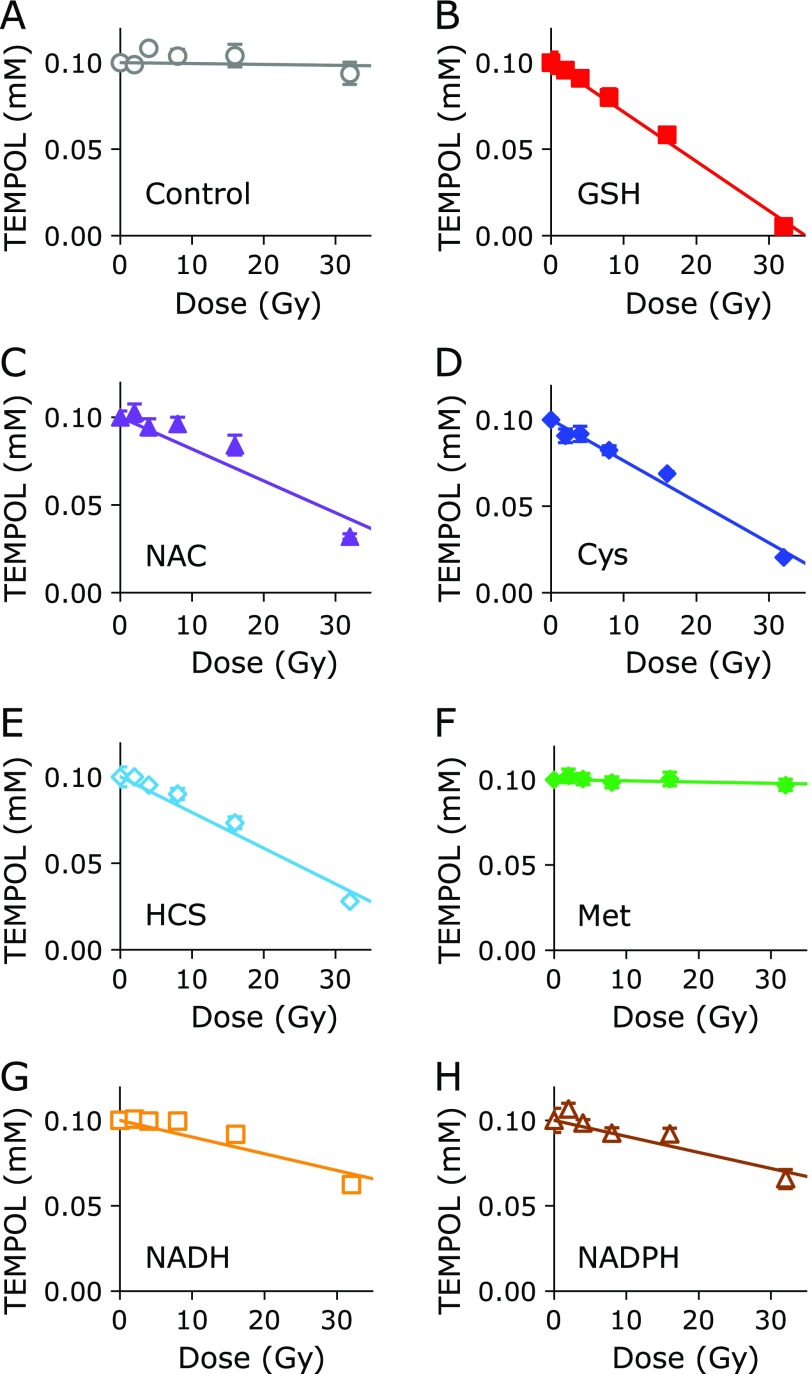
X-ray dose-dependent TEMPOL decay with a coexisting test compound. (A) TEMPOL alone. TEMPOL with (B) GSH, (C) NAC, (D) Cys, (E) HCS, (F) Met, (G) NADH, or (H) NADPH. The data points and error bars indicate the average ± SD of 3 experiments. The concentrations of TEMPOL and the test compound were 0.1 mM and 1.0 mM, respectively.

The X-ray dose-dependent TEMPOL reduction profiles with coexisting test compounds in PB are shown in Fig. 3. X-ray induced ^•^OH and/or HO_2_^•^, which can also one-electron-oxidize TEMPOL to produce the oxoammonium cation form. Almost no reduction of TEMPOL was observed when TEMPOL alone was irradiated with X-rays (Fig. 3A). All thiol compounds tested here, including GSH, NAC, Cys, and HCS, reduced TEMPOL during X-ray irradiation (Fig. 3B–E). However, coexisting Met was unable reduce TEMPOL during X-ray irradiation (Fig. 3F). Coexisting NADH or NADPH reduced TEMPOL during X-ray irradiation, but at lower levels than the thiols (Fig. 3G and H).

X-ray irradiation of water molecules (H_2_O) generates ^•^OH and ^•^H due to the radiolysis of H_2_O. ^•^OH itself is a strong oxidant and can one-electron oxidize TEMPOL to produce the oxoammonium cation form. On the other hand, ^•^H can react with dissolved oxygen to produce HO_2_^•^, which can one-electron oxidize TEMPOL. When a hydrogen donor, such as thiol compounds or NAD(P)H, coexist with the oxoammonium cation form of TEMPOL, the oxoammonium cation can receive hydrogen and become the corresponding hydroxylamine form, which is the two-electron reduced form of the oxoammonium cation form or one-electron reduced form of the nitroxyl radical form. Separate from the above reaction, oxoammonium cations can directly react with thiols to make a complex. If no hydrogen donors or thiols coexist in the reaction system, oxoammonium cations rapidly one-electron reduce back to nitroxyl radicals, and no notable change is observed.

**Fig. 4:**
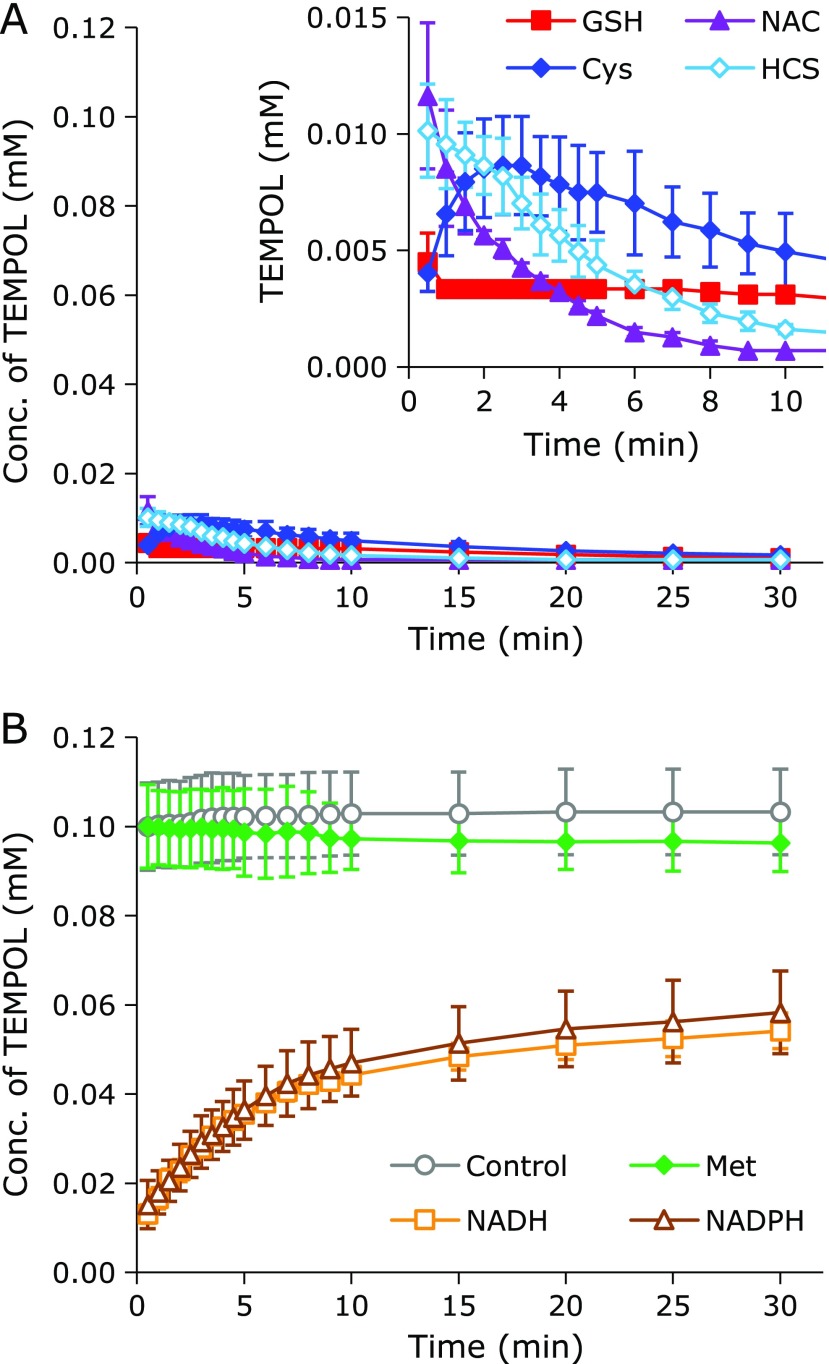
Time course of TEMPOL in the ferricyanide reaction system with a coexisting test compound. (A) Reaction profiles of TEMPOL in the ferricyanide reaction system with a thiol compound, closed square: GSH, closed triangle: NAC, closed diamond: Cys, and open diamond: HCS. (B) Reaction profiles of TEMPOL in the ferricyanide reaction system with a non-thiol compound, closed circle: Met, open square: NADH, and open triangle: NADPH. The open circles indicate the reaction profile of TEMPOL alone in the ferricyanide reaction system. The data points and error bars indicate the average ± SD of 3 experiments. The concentrations of TEMPOL and the test compound were 0.1 mM and 1.0 mM, respectively. The insert in (A) is an enlarged view of the time window of 0–10 min.

The reaction profiles of TEMPOL with the test compound in PB when potassium ferricyanide {K_3_[Fe(CN)_6_]} was added are shown in Fig. 4. All thiol compounds were able to reduce TEMPOL immediately after the addition of ferricyanide (Fig. 4A). The behaviors of thiols in this reaction system were followed carefully when closing the reaction profiles (Fig. 4A, insertion). The reaction profile with coexisting Cys exhibited a perceptible recovery phase of TEMPOL, i.e., re-oxidation of the hydroxylamine form to the nitroxyl radical form. Although the reaction profiles with coexisting GSH or HCS did not include such a clear recovery phase, an equilibrium state or slight bulging was observed on the decay slope. These may be traces of the recovery phase of TEMPOL. TEMPOL decay with coexisting NAC almost fit simple first-order decay.

[Fe^III^(CN)_6_]^3−^ oxidizes TEMPOL to the oxoammonium cation, then [Fe^III^(CN)_6_]^3−^ becomes [Fe^II^(CN)_6_]^4−^. The [Fe^II^(CN)_6_]^4−^ ion can reduce the oxoammonium cation back to TEMPOL. TEMPOL alone remained stable with ferricyanide (Fig. 4B, open circle). Met (Fig. 4B, closed circle) was non-reactive in this system. NADH (Fig. 4B, open triangle) and NADPH (Fig. 4B, open square) quickly reduced TEMPOL, whose levels recovered gradually. This suggested that NAD(P)H mainly functions as a hydrogen donor to change the oxoammonium cation into the hydroxylamine form, which can be re-oxidized to recover TEMPOL with excess [Fe^III^(CN)_6_]^3−^ ions. On the other hand, because excess [Fe^III^(CN)_6_]^3−^ ions were unable to restore TEMPOL, thiol compounds mainly react directly with oxoammonium cations to generate a stable complex. Thiols can also function as hydrogen donors and reduce a fraction of TEMPOL to the corresponding hydroxylamine form (i.e., TEMPOL-H) because the recovery phase or trace of recovery was observed in the reaction profiles of TEMPOL, as shown in Fig. 4A.

**Fig. 5:**
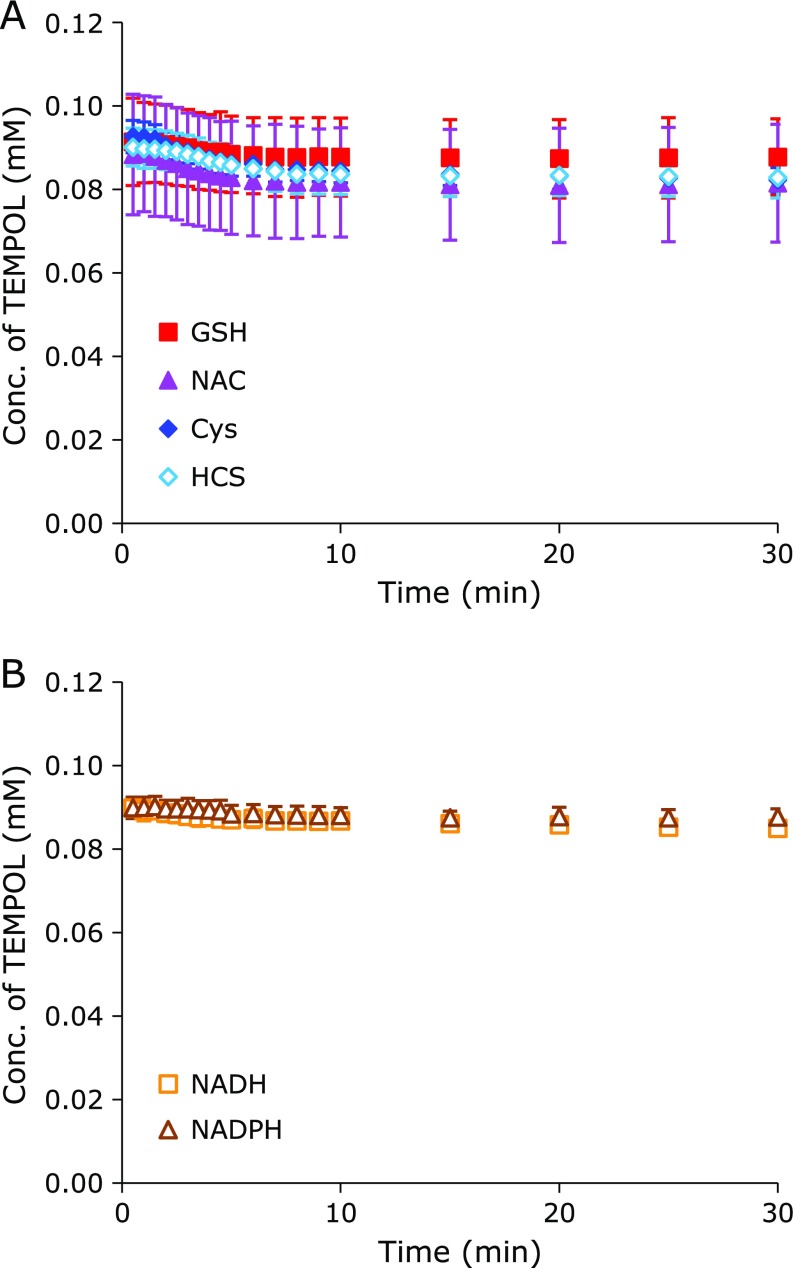
Time course of TEMPOL in the FeCl_3_ reaction system with a coexisting test compound. (A) Reaction profiles of TEMPOL in the FeCl_3_ reaction system with a thiol compound, closed square: GSH, closed triangle: NAC, closed diamond: Cys, and open diamond: HCS. (B) Reaction profiles of TEMPOL in the FeCl_3_ reaction system with a non-thiol compound, open square: NADH, and open triangle: NADPH. The marks and error bars indicate the average ± SD of 3 experiments.

Addition of FeCl_3_ to the corresponding reaction mixture instead of K_3_[Fe(CN)_6_] did not result in such reactions of TEMPOL (Fig. 5). The free Fe^3+^ ion may not oxidize TEMPOL to the corresponding oxoammonium cation. The free Fe^3+^ ion was not reduced by thiol compounds, i.e., free Fe^3+^ ions were unable to oxidize thiol compounds (data not shown). In addition, FeCl_3_ was unable to oxidize TEMPOL-H to TEMPOL (data not shown). Although FeCl_3_ has been widely used as an inducer of oxidative stress *in vivo* or in cell culture experiments, the direct reaction of free Fe^3+^ and organic compounds is mild.

**Fig. 6:**
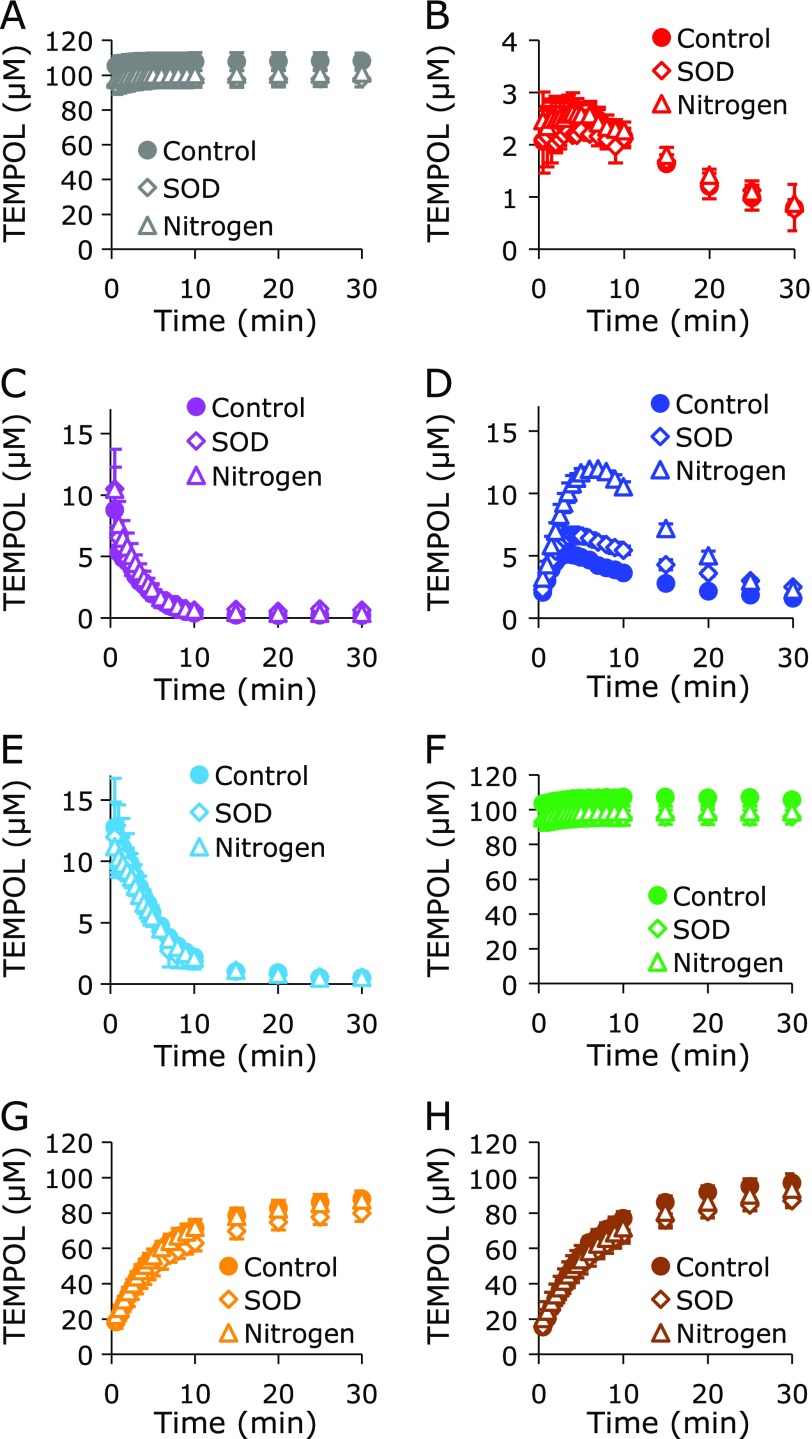
Effects of SOD and N_2_-bubbling on the time course of TEMPOL in the ferricyanide reaction system with a coexisting test compound. (A) Reaction profiles of TEMPOL in the ferricyanide reaction system with no other reductant. Reaction profiles of TEMPOL in the ferricyanide reaction system with (B) GSH, (C) NAC, (D) Cys, (E) HCS, (F) Met, (G) NADH, or (H) NADPH. The marks and error bars indicate the average ± SD of 3 experiments.

The effects of SOD or N_2_-bubbling on ferricyanide-induced TEMPOL decay are shown in Fig. 6. Neither SOD nor N_2_-bubbling suppressed the initial rapid TEMPOL decay. This suggests that the ferricyanide-induced rapid TEMPOL decay with the coexisting thiol compound was not related to oxygen-related free radicals. At the same time, the temporal recovery of TEMPOL after the initial rapid decay with coexisting Cys was increased by SOD or N_2_-bubbling (Fig. 6D). N_2_-bubbling increased temporal TEMPOL recovery with coexisting Cys more than SOD. The temporal recovery of TEMPOL observed with coexisting GSH was not affected by SOD or N_2_-bubbling (Fig. 6B). This suggests that oxygen-related free radicals are related, to some degree, to the relatively slow second TEMPOL decay observed with coexisting Cys.

**Fig. 7:**
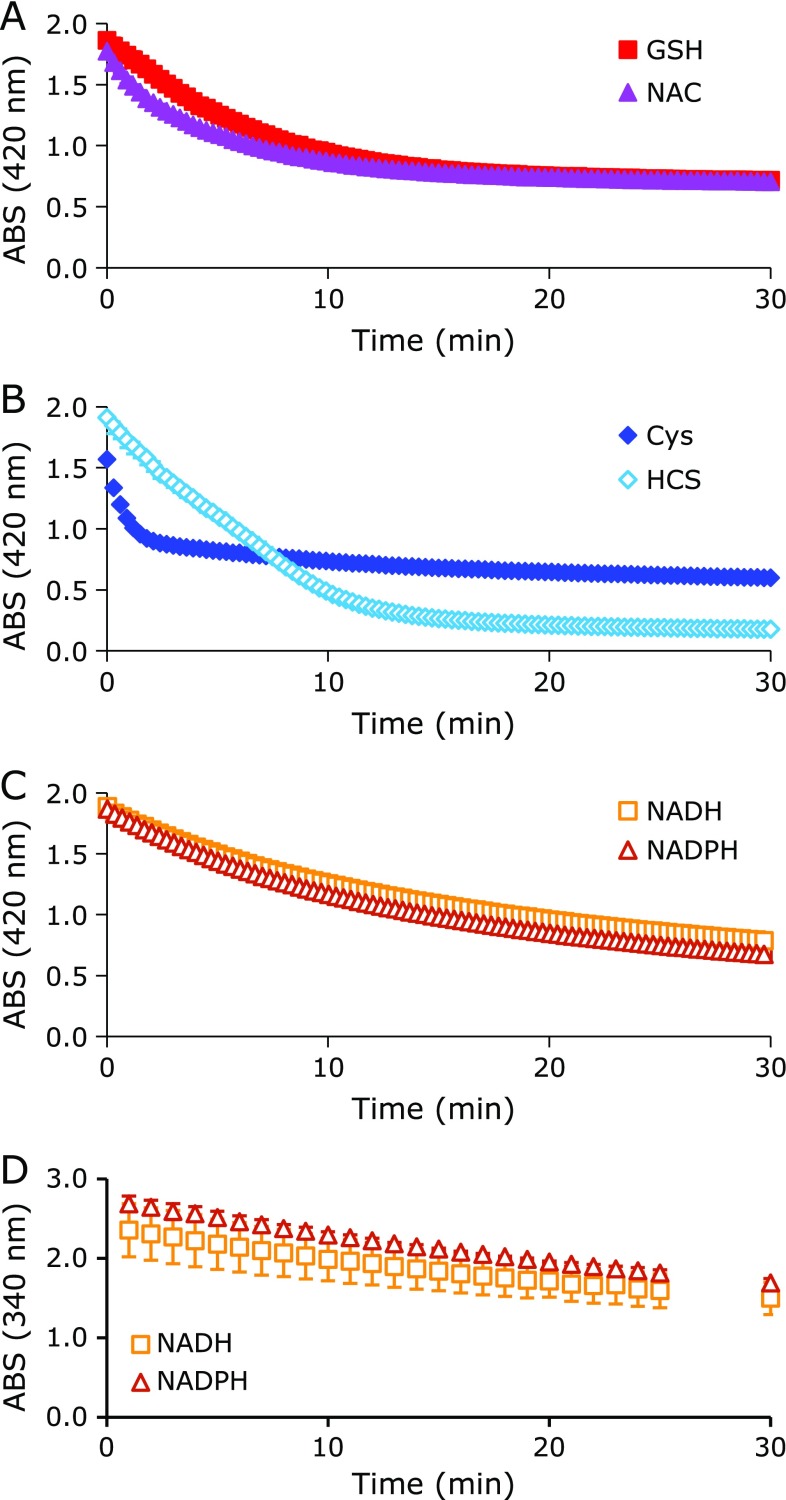
Reduction time course of ferricyanide by a thiol compound or NAD(P)H. (A) Reduction profiles of ferricyanide by GSH (closed square) or NAC (closed triangle). (B) Reduction profiles of ferricyanide by Cys (closed diamond) or HCS (open diamond). (C) Reduction profiles of ferricyanide by NADH (open square) or NADPH (open triangle). (D) Oxidation profiles of NADH (open square) or NADPH (open triangle) by ferricyanide. The marks and error bars indicate the average ± SD of 3 experiments. The concentrations of ferricyanide and test compound were 2 mM and 1 mM, respectively.

The redox potential of TEMPOL vs the corresponding oxoammonium cation form was reported to be +0.810 V.^(21)^ It may be difficult for K_3_[Fe(CN)_6_] to oxidize TEMPOL to the oxoammonium cation form based on its redox potential, which was reported to be +0.410 V.^(21)^ The thiol compounds examined reduced K_3_[Fe(CN)_6_], i.e., K_3_[Fe(CN)_6_] oxidized thiol compounds, although the reaction was slow (Fig. 7A). The rapid TEMPOL decay induced by K_3_[Fe(CN)_6_] also occurred with coexisting NAD(P)H. However, the reduction of K_3_[Fe(CN)_6_] by NAD(P)H and the oxidation reaction of NAD(P)H by K_3_[Fe(CN)_6_] were also slow (Fig. 7B and C). The addition of 0.1 mM TEMPOL to the reaction mixture quickened the reduction of K_3_[Fe(CN)_6_] by NAD(P)H, but this cannot explain the K_3_[Fe(CN)_6_]-induced rapid TEMPOL decay, as shown in Fig. 4. Therefore, complex enzyme-like catalytic oxidative reactions may have occurred with K_3_[Fe(CN)_6_], for which the detailed mechanisms are unclear. The [Fe^III^(CN)_6_]^3−^ ion may function as a strong oxidant when coexisting with a hydrogen donor, and can one-electron-oxidize TEMPOL to produce the oxoammonium cation form.

As shown in Fig. 2–4, in a reaction mixture containing TEMPOL and a thiol compound in an oxidative atmosphere, TEMPOL was reduced. TEMPOL can be oxidized to the oxoammonium cation form and then the oxoammonium cation can then be reduced by the thiol. The oxoammonium cation may be reduced by either ^•^H or the companion thiyl radical. The reduction of TEMPOL coexisting with a thiol compound suggests the generation of oxidants in the reaction mixture.

Based on the results of experiments shown in Fig. 2–4, we formed the hypothesis that the generation/addition of an oxidant in the reaction mixture containing TEMPOL and a thiol compound can reduce TEMPOL. If this is true, the generation of the O_2_^•−^ and HO_2_^•^ redox pair, independent of its source, in a reaction mixture containing TEMPOL and a thiol compound can reduce TEMPOL. We previously reported HO_2_^•^/O_2_^•−^ generation by the reduction of resolved O_2_ in an aqueous solution containing GSH at a hyperthermal temperature.^(5,6)^ When the thiol compounds assessed in this paper, i.e., GSH, Cys, HCS, and NAC, reduced molecular oxygen, the reduction of TEMPOL in the reaction mixture was observed.

**Fig. 8:**
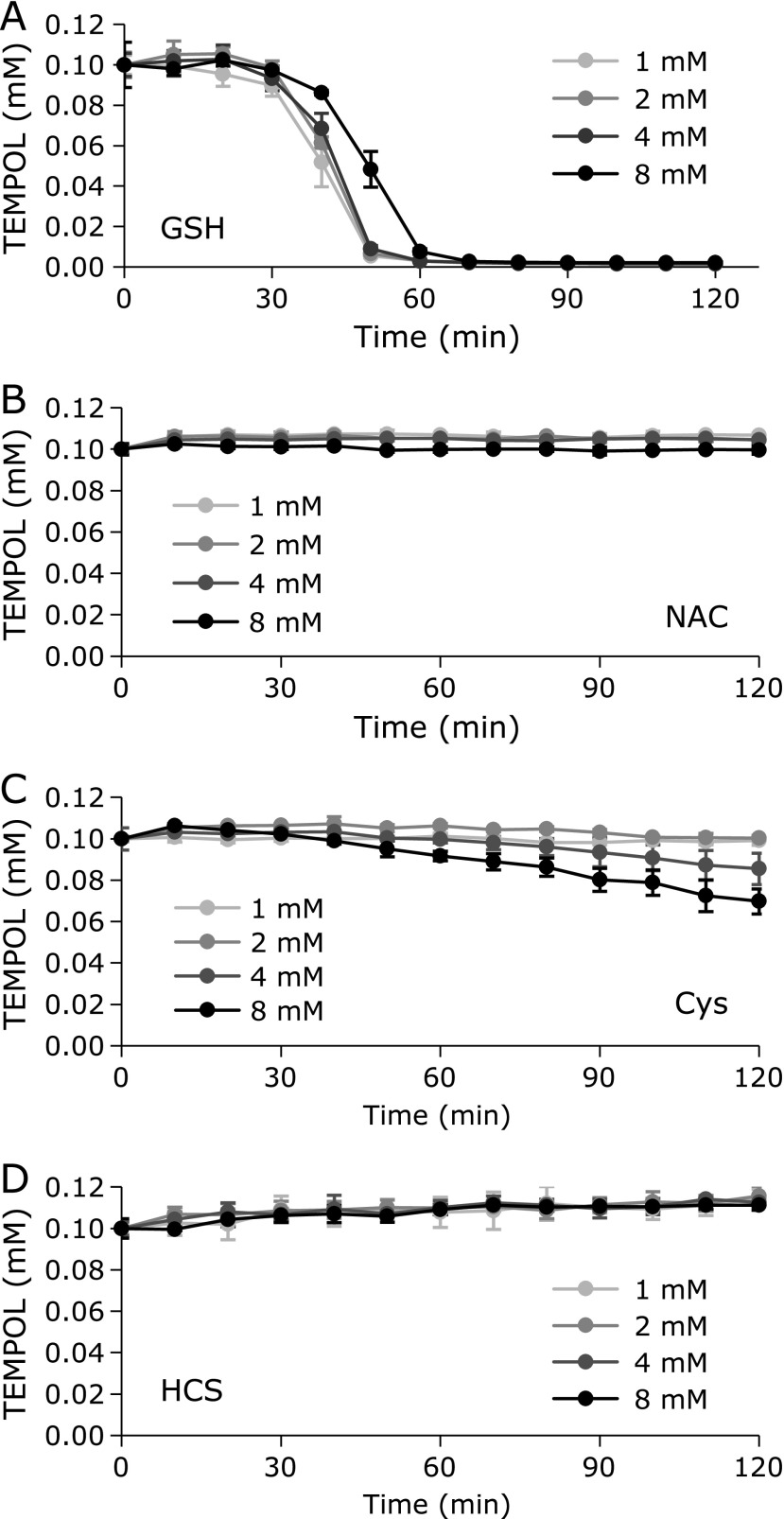
Time course of TEMPOL with coexisting thiol compounds at a hyperthermal temperature. Reaction profiles of TEMPOL with (A) GSH, (B) NAC, (C) Cys, or (D) HCS. The data points and error bars indicate the average ± SD of 3 experiments. The concentration of TEMPOL was 0.1 mM. Several concentrations (1.0, 2.0, 4.0, or 8.0 mM) of the thiol compound were tested.

The reaction profiles when TEMPOL was incubated with thiols at a hyperthermal temperature (44°C) are shown in Fig. 8. As reported previously, GSH caused the EPR signal decay of TEMPOL with a characteristic profile of a time delay and sequential steep decay (Fig. 8A). GSH-dependent TEMPOL decay was slightly delayed after increasing the GSH concentration. NAC, however, caused no EPR signal decay (Fig. 8B). Cys caused very slow decay of TEMPOL. The Cys-induced TEMPOL reduction increased with increasing Cys concentration (Fig. 8C). HCS induced no EPR signal decay of TEMPOL after hyperthermal treatment (Fig. 8D). Methionine, which is not a thiol, caused no EPR signal decay of TEMPOL after hyperthermal treatment (data not shown).

The order of reduction activity of the thiols for O_2_ based on TEMPOL reduction was GSH > Cys > HCS ≈︀ NAC. However, the reduction activity of thiols versus DPPH radicals was Cys > HCS > GSH ≈︀ NAC, which was ordered according to the p*K*a of the thiol moiety.^(22)^ The order of reducing activity predicted from the results shown in Fig. 2 was similar to the reduction activity of thiols for DPPH. The reduction activity of thiols for O_2_ is not necessarily the same as that for DPPH. The apparent reductive activity may change due to environment-dependent reaction mechanisms such as electron transfer, hydrogen transfer, or other combinations.

**Fig. 9:**
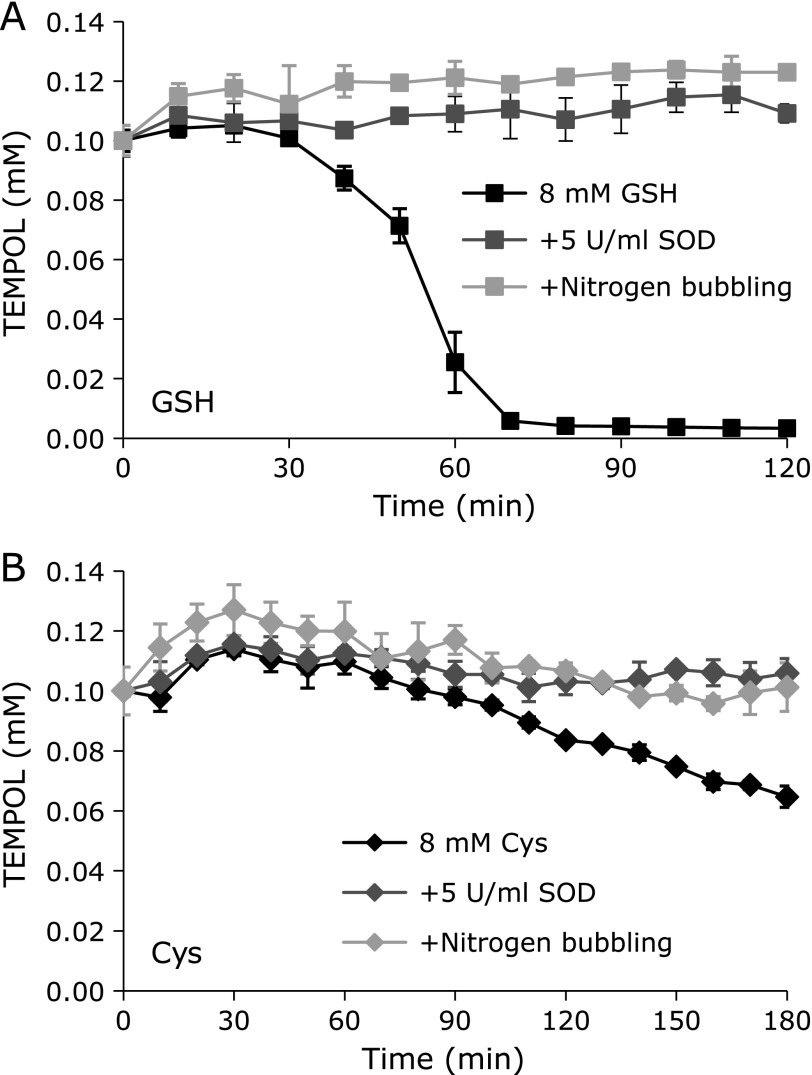
Effects of SOD and N_2_ gas aeration on GSH- and Cys-induced TEMPOL decay at a hyperthermal temperature. Reaction profiles of TEMPOL with coexisting (A) GSH or (B) Cys. The data points and error bars indicate the average ± SD of 3 experiments. The concentrations of TEMPOL and thiols were 0.1 mM and 8.0 mM, respectively. Dark gray marks indicate the reaction profiles with 5 U/ml of SOD in the reaction mixture. Light gray marks indicate the reaction profiles with N_2_ gas aeration of the reaction mixture.

GSH-induced and Cys-induced TEMPOL decay at 44°C were inhibited by the addition of SOD and/or by bubbling N_2_ gas into the reaction mixture (Fig. 9). Therefore Cys-induced TEMPOL decay was also mediated by HO_2_^•^/O_2_^•−^ generation, as reported for GSH.^(5)^ Thus, GSH and Cys can reduce molecular oxygen to make HO_2_^•^/O_2_^•−^, even though Cys-induced HO_2_^•^/O_2_^•−^ generation was slow. HCS cannot generate O_2_^•−^ in a direct manner, whereas HCS-induced oxidative stress is strongly related to the *in vivo* generation of O_2_^•−^. Thus, chemically, HCS is a reductant.

As we reported previously,^(5,6)^ the reduction of TEMPOL with coexisting GSH was temperature dependent and increased at higher temperatures. Indeed the reduction of molecular oxygen by GSH was facilitated by higher temperatures. The generation of GS^•^ may induce the reduction O_2_, but the detail mechanism for the provision of GS^•^ remains unclear because only the ability of other thiol compounds to reduce O_2_ was assessed in this study.

The reduction of TEMPOL by coexisting GSH was delayed with increasing concentrations of GSH (Fig. 8A), as reported previously.^(5)^ This suggested that HO_2_^•^ can react with GSH competitively with TEMPOL because higher concentrations of GSH were able to slightly suppress the one-electron-oxidation of TEMPOL to the oxoammonium form, even if HO_2_^•^ generation from O_2_ increased at higher concentrations of GSH. However, Cys may reduce HO_2_^•^ at a sufficiently fast rate to suppress the reduction of TEMPOL at lower concentrations of Cys, which may result in lower concentrations of HO_2_^•^ by reducing dissolved O_2_ than GSH. Due to the increase in TEMPOL reduction by coexisting Cys with increasing Cys concentration (Fig. 8C), HO_2_^•^ generation by reducing O_2_ may simply increase with increasing concentrations of Cys. Once an oxidative atmosphere is created by HO_2_^•^ generation in the reaction mixture, the EPR signal of TEMPOL may begin decreasing.

There are numerous reports on ROS generation in cells and animal models under hyperthermal conditions.^(23–25)^ Local hyperthermia may easily occur under non-physiological conditions, even during regular daily activities such as using a hair dryer, disposable body warmer, or taking a hot bath. Thus, researchers should take measures to make comfortable (less stressful) experimental conditions for animals such as controlling the body temperature under anesthesia during functional imaging experiments. Even at a physiological temperature, i.e., 37°C, more than a 2-h incubation with GSH and O_2_ may cause oxidative stress with increased ROS levels.^(6)^ Therefore, temperature-dependent and GSH-induced HO_2_^•^/O_2_^•−^ generation are considered causes of cell death.

EPR signal loss by TEMPOL with coexisting thiol compounds in an aqueous reaction mixture can occur after several reactions. An oxidant may directly oxidize a thiol to produce a thiyl radical (RS^•^), and then the RS^•^ can react directly with the nitroxyl radical (>N-O^•^). Goldstein *et al.*^(12)^ reported that RS^•^ and the nitroxyl radical >N-O^•^ directly react, and make a complex compound (>N-O-SR). Then, >N-O-SR becomes a stable amine form (>NH). They also proposed two alternate methods to produce >N-O-SR either by the reaction of an oxoammonium cation (>N^+^=O) and thiolate (RS^−^), which produces >N-O-SR directly, or by reacting >N-O^•^ and RS^•^, which then produce >N-O-SR. In this study, an important requirement was the irreversibleness of the reaction from >N-O^•^ to >N-O-SR. When oxidation of the hydroxylamine form (>N-OH) to the corresponding >N-O^•^ form, i.e., recovery of the EPR signal, was observed, it indicated the reduction of >N-O^•^ to >N-OH. In other words, >N^+^=O received hydrogen from the thiols (Fig. 4 and 6). As the reactions of thiol compounds and K_3_[Fe(CN)_6_] were not fast (Fig. 7), and rapid TEMPOL decay was observed with coexisting NAD(P)H after adding K_3_[Fe(CN)_6_] (Fig. 4B), the initial RS^•^ generation by an oxidant may be not the main reaction.

Biologically reactive sulfur species, typified by thiols, mediate many pathophysiologically important redox reactions, termed Red-S-Ox. We demonstrated reductive stress caused by thiol compounds such as GSH and/or Cys.

## Conclusion

Thiols and NAD(P)H can react with TEMPOL under oxidative conditions such as in an HX-XO reaction system, after X-ray irradiation, or in a ferricyanide reaction system. Thiols can react with the oxoammonium cation to make an EPR silent complex and function as an H-donor to provide the hydroxylamine. GSH and Cys can reduce molecular oxygen to generate HO_2_^•^/O_2_^•−^, whereas Cys-induced HO_2_^•^/O_2_^•−^ production was slow. Therefore, GSH and Cys can cause reductive stress. NAC, which was unable to reduce TEMPOL at hyperthermal temperatures, is a simple tractable antioxidant.
